# The genome sequence of a robberfly *Leptogaster cylindrica* (De Geer, 1776)

**DOI:** 10.12688/wellcomeopenres.22761.1

**Published:** 2024-08-12

**Authors:** Olga Sivell, Duncan Sivell, Natural History Museum Genome Acquisition Lab, Darwin Tree of Life Barcoding collective, Wellcome Sanger Institute Tree of Life Management, Samples and Laboratory team, Wellcome Sanger Institute Scientific Operations: Sequencing Operations, Wellcome Sanger Institute Tree of Life Core Informatics team, Tree of Life Core Informatics collective, Darwin Tree of Life Consortium

**Affiliations:** 1 Natural History Museum, London, England, UK

**Keywords:** Leptogaster cylindrica, robberfly, genome sequence, chromosomal, Diptera

## Abstract

We present a genome assembly from an individual female *Leptogaster cylindrica* (robberfly; Arthropoda; Insecta; Diptera; Asilidae). The genome sequence spans 196.60 megabases. Most of the assembly is scaffolded into 5 chromosomal pseudomolecules. The mitochondrial genome has also been assembled and is 18.0 kilobases in length. Gene annotation of this assembly on Ensembl identified 10,816 protein-coding genes.

## Species taxonomy

Eukaryota; Opisthokonta; Metazoa; Eumetazoa; Bilateria; Protostomia; Ecdysozoa; Panarthropoda; Arthropoda; Mandibulata; Pancrustacea; Hexapoda; Insecta; Dicondylia; Pterygota; Neoptera; Endopterygota; Diptera; Brachycera; Muscomorpha; Asiloidea; Asilidae; Leptogastrinae; *Leptogaster*; *Leptogaster cylindrica* (De Geer, 1776) (NCBI:txid468752).

## Background

*Leptogaster cylindrica* is a slender fly from the family Asilidae (robberflies). It is a relatively small robberfly, greyish in colour, with mostly yellow legs. It can be separated from the similar looking *L. guttiventris* by the continuous black line on tergites 4 and 5, which is interrupted in *L. guttiventris*. The proportions of the second sternite also differ in these two species, with the basal section being 1.5 times as long as it is wide in *L. cylindrica*, while it is twice as long as it is wide in *L. guttiventris*. Both species are very variable in size. In *L. cylindrica* the body length ranges from 6 to 15 mm and the wing length from 4 to 8 mm. There are other European species of similar appearance, although they have not been recorded from Britain ( Stubbs & Drake, 2014).

*Leptogaster cylindrica* is a Palaearctic species ( Lehr, 1988). In Britain this species’ distribution is mainly southern England, becoming scarcer further north, with a few sites (mainly coastal) in Wales. In recent years it has been recorded from Scotland, with the most northerly record from Tentsmuir in north-east Fife ( Soldierflies and Allies Recording Scheme, 2023). Its preferred habitats are open grasslands on fairly dry soils, and also road verges. The flight period is from mid-May to August, with a peak in late June and mid-July ( Stubbs & Drake, 2014).

*Leptogaster cylindrica* is predatory, hunting flies and other small insects. It occasionally captures spiders. It flies close to the ground among vegetation, particularly tall grass, grabbing resting prey ( Dennis *et al.*, 2012). The prey is immobilised or killed instantly using venom injected through a syringe-like hypopharynx ( Walker *et al.*, 2018; Whitfield, 1925). The females of *Leptogaster* species lay eggs in flight or while resting on vegetation, dropping them singly to the ground. Between 1 and 8 eggs are laid in this manner during an oviposition event. The eggs hatch after 9–14 days. The larvae are soil dwelling ( Dennis *et al.*, 2013). The larva and pupa have been described by Melin (1923)

This genome is based on a single specimen of *Leptogaster cylindrica* (NHMUK014111064) collected by O. Sivell from Wigmore Park, Luton on 17/06/2020. It was identified by D. Sivell following Stubbs and Drake (2014). The phylogeny of Asilidae has been studied by Dikow ( 2009a; 2009b), who also provided some genomic and transcriptomic resources for the family, including a complete genome of *Proctacanthus coquilletti* Hine, 1911 ( Dikow *et al.*, 2017). We believe that the complete genome of *Leptogaster cylindrica* presented here will aid research into the phylogeny and biology of Asilidae.

## Genome sequence report

**Figure 1.**
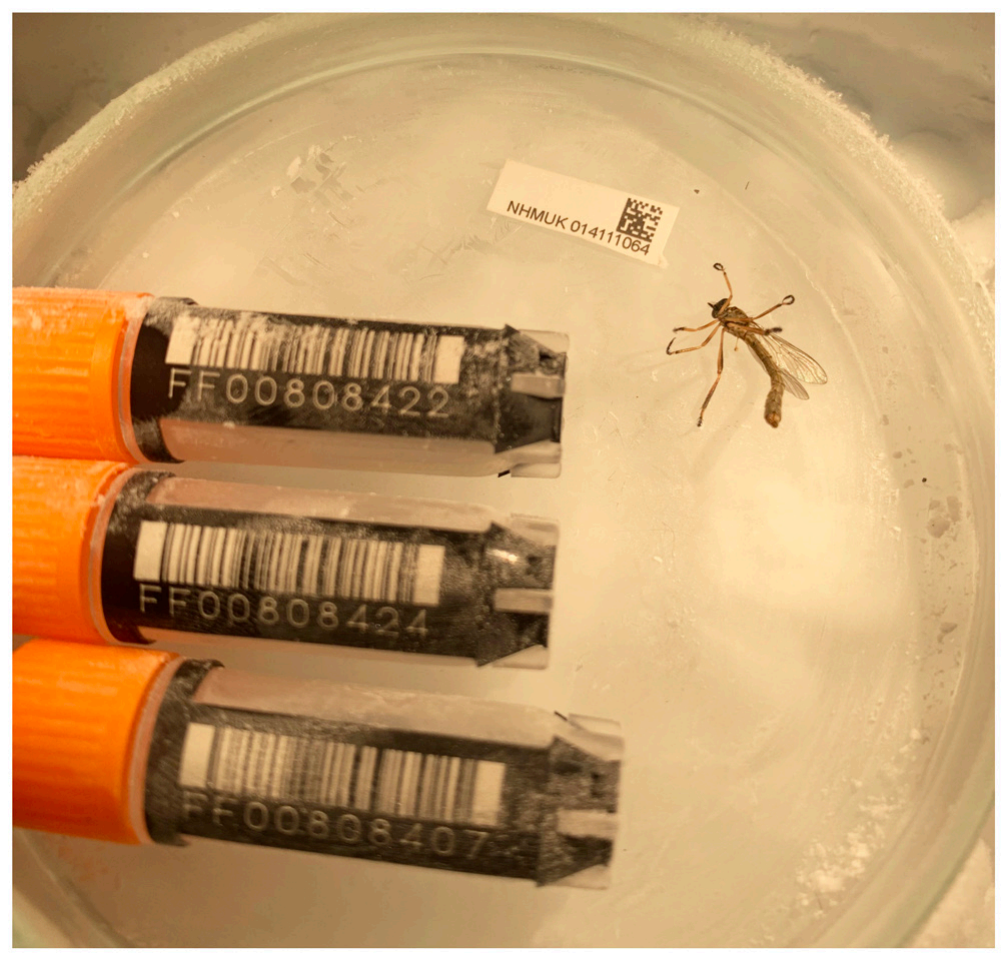
Photograph of the *Leptogaster cylindrica* (idLepCyli1) specimen used for genome sequencing.

The genome of an adult female *Leptogaster cylindrica* ( Figure 1) was sequenced using Pacific Biosciences single-molecule HiFi long reads, generating a total of 15.97 Gb (gigabases) from 1.64 million reads, providing approximately 86-fold coverage. Primary assembly contigs were scaffolded with chromosome conformation Hi-C data, which produced 104.59 Gbp from 692.64 million reads, yielding an approximate coverage of 532-fold. Specimen and sequencing information is summarised in Table 1.

**Table 1.** Specimen and sequencing data for *Leptogaster cylindrica*.

Project information			
**Study title**	Leptogaster cylindrica		
**Umbrella BioProject**	PRJEB57890		
**Species**	*Leptogaster cylindrica*		
**BioSample**	SAMEA7521500		
**NCBI taxonomy ID**	468752		
**Specimen information**			
**Technology**	**ToLID**	**BioSample accession**	**Organism part**
**PacBio long read sequencing**	idLepCyli1	SAMEA7521537	thorax
**Hi-C sequencing**	idLepCyli1	SAMEA7521536	head
**RNA sequencing**	idLepCyli3	SAMEA7521560	thorax
**Sequencing information**			
**Platform**	**Run accession**	**Read count**	**Base count (Gb)**
**Hi-C Illumina NovaSeq 6000**	ERR10614870	6.93e+08	104.59
**PacBio Sequel IIe**	ERR10662016	1.64e+06	15.97
**RNA Illumina HiSeq 4000**	ERR10614871	4.54e+07	6.85

**Figure 2.**
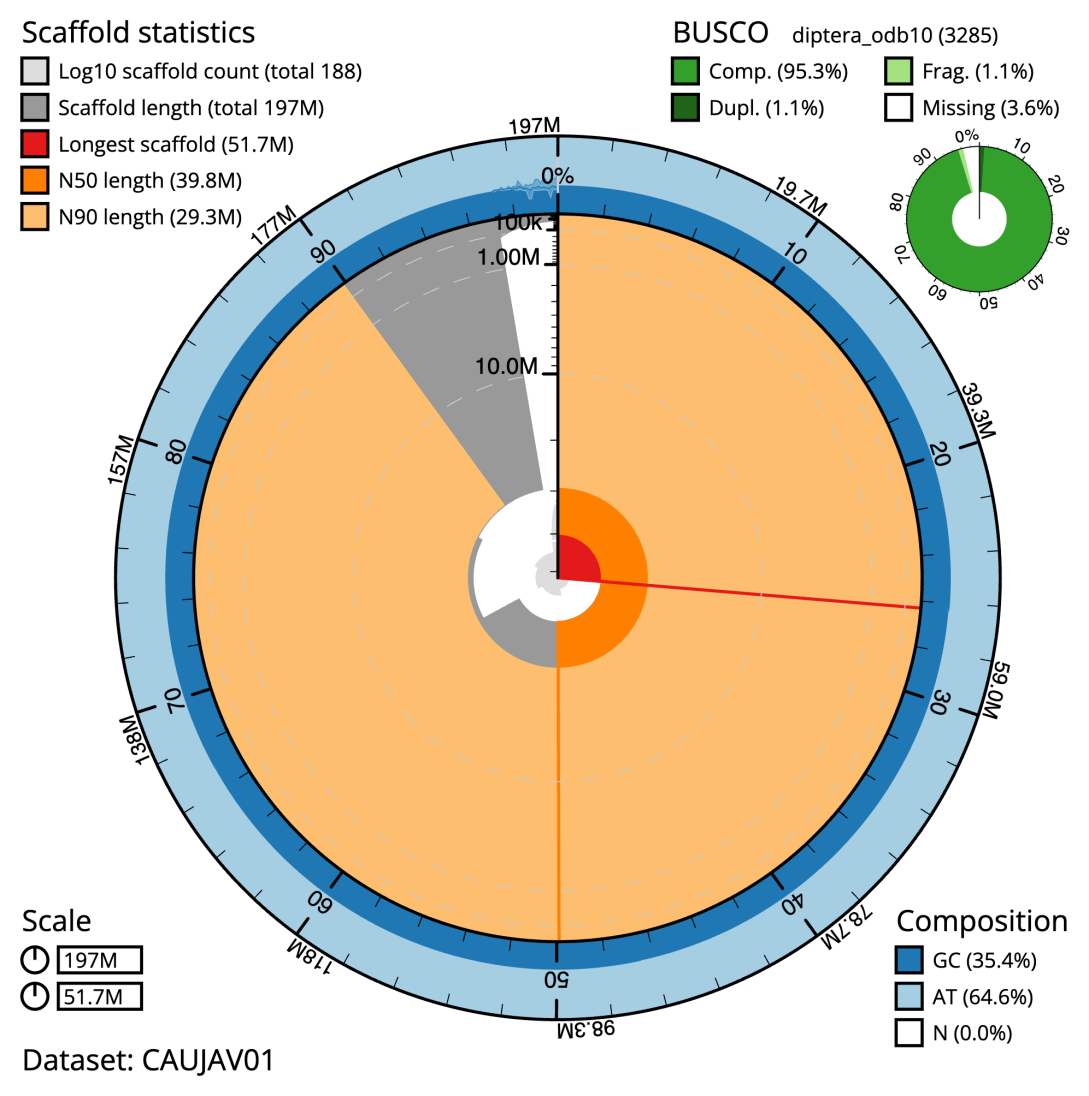
Genome assembly of *Leptogaster cylindrica*, idLepCyli1.1: metrics. The BlobToolKit snail plot shows N50 metrics and BUSCO gene completeness. The main plot is divided into 1,000 size-ordered bins around the circumference with each bin representing 0.1% of the 196,645,336 bp assembly. The distribution of scaffold lengths is shown in dark grey with the plot radius scaled to the longest scaffold present in the assembly (51,725,364 bp, shown in red). Orange and pale-orange arcs show the N50 and N90 scaffold lengths (39,823,257 and 29,318,419 bp), respectively. The pale grey spiral shows the cumulative scaffold count on a log scale with white scale lines showing successive orders of magnitude. The blue and pale-blue area around the outside of the plot shows the distribution of GC, AT and N percentages in the same bins as the inner plot. A summary of complete, fragmented, duplicated and missing BUSCO genes in the diptera_odb10 set is shown in the top right. An interactive version of this figure is available at https://blobtoolkit.genomehubs.org/view/Leptogaster%20cylindrica/dataset/CAUJAV01/snail.

**Figure 3.**
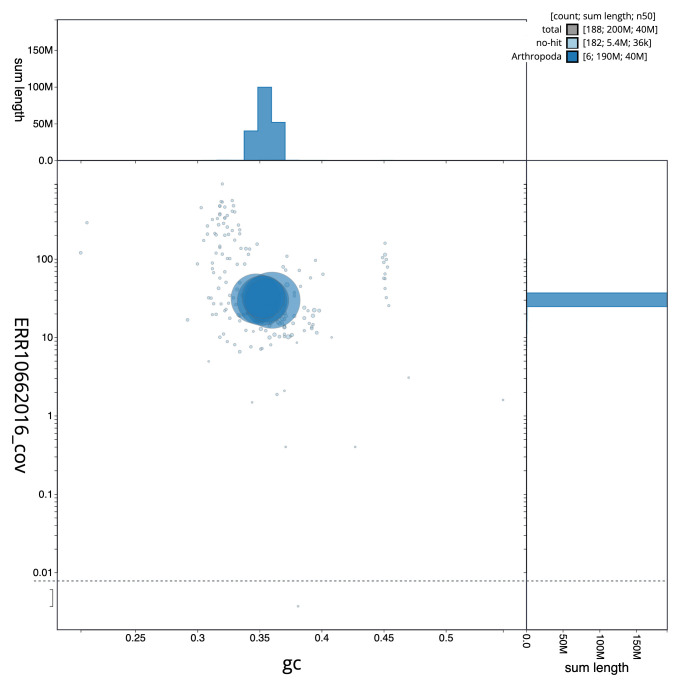
Genome assembly of *Leptogaster cylindrica*, idLepCyli1.1: BlobToolKit GC-coverage plot. Sequences are coloured by phylum. Circles are sized in proportion to sequence length. Histograms show the distribution of sequence length sum along each axis. An interactive version of this figure is available at https://blobtoolkit.genomehubs.org/view/Leptogaster%20cylindrica/dataset/CAUJAV01/blob.

**Figure 4.**
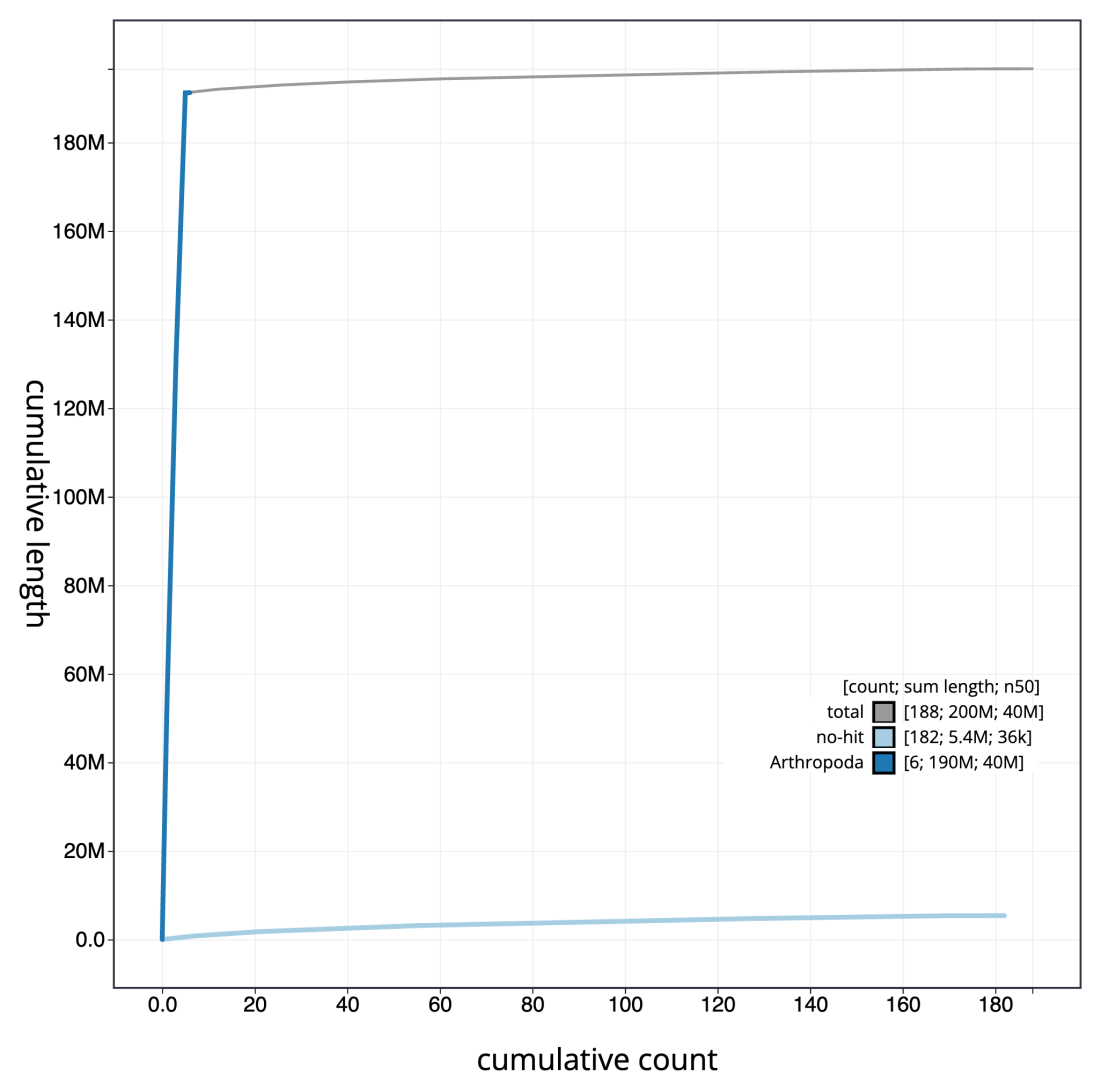
Genome assembly of *Leptogaster cylindrica* idLepCyli1.1: BlobToolKit cumulative sequence plot. The grey line shows cumulative length for all sequences. Coloured lines show cumulative lengths of sequences assigned to each phylum using the buscogenes taxrule. An interactive version of this figure is available at https://blobtoolkit.genomehubs.org/view/Leptogaster%20cylindrica/dataset/CAUJAV01/cumulative.

**Figure 5.**
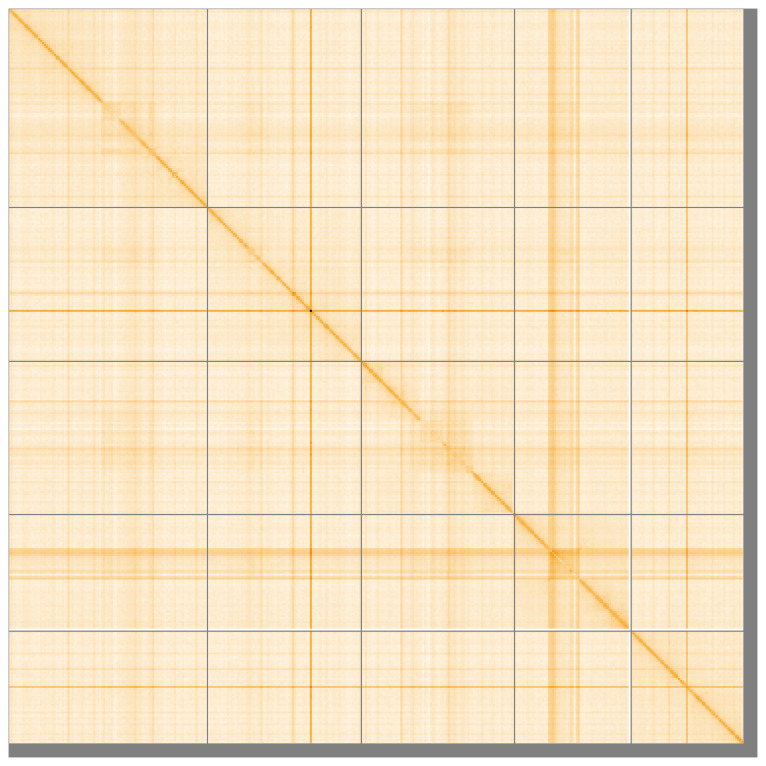
Genome assembly of *Leptogaster cylindrica* idLepCyli1.1: Hi-C contact map of the idLepCyli1.1 assembly, visualised using HiGlass. Chromosomes are shown in order of size from left to right and top to bottom. An interactive version of this figure may be viewed at https://genome-note-higlass.tol.sanger.ac.uk/l/?d=Qhgr_OTRSx6CA29r_IkzLQ.

Manual assembly curation corrected 179 missing joins or mis-joins and 5 haplotypic duplications, reducing the assembly length by 0.82% and the scaffold number by 34.49%, and increasing the scaffold N50 by 76.41%. The final assembly has a total length of 196.60 Mb in 187 sequence scaffolds with a scaffold N50 of 39.8 Mb ( Table 2). The total count of gaps in the scaffolds is 288. The snail plot in Figure 2 provides a summary of the assembly statistics, while the distribution of assembly scaffolds on GC proportion and coverage is shown in Figure 3. The cumulative assembly plot in Figure 4 shows curves for subsets of scaffolds assigned to different phyla. Most (97.26%) of the assembly sequence was assigned to 5 chromosomal-level scaffolds. Chromosome-scale scaffolds confirmed by the Hi-C data are named in order of size ( Figure 5; Table 3). The exact order and orientation of the contigs in the centromeric repeat regions of chromosome 3 (13.3–29.5 Mb) and chromosome 4 (9.4–17.4 Mb) is unknown. No sex chromosome could be identified. The coverage suggests that the specimen is a homogametic XX female sample. While not fully phased, the assembly deposited is of one haplotype. Contigs corresponding to the second haplotype have also been deposited. The mitochondrial genome was also assembled and can be found as a contig within the multifasta file of the genome submission.

**Table 2.** Genome assembly data for *Leptogaster cylindrica*, idLepCyli1.1.

Genome assembly		
Assembly name	idLepCyli1.1	
Assembly accession	GCA_963082835.1	
*Accession of alternate haplotype*	*GCA_963082695.1*	
Span (Mb)	196.60	
Number of contigs	476	
Contig N50 length (Mb)	1.4	
Number of scaffolds	187	
Scaffold N50 length (Mb)	39.8	
Longest scaffold (Mb)	51.73	
Assembly metrics *		*Benchmark*
Consensus quality (QV)	60.2	*≥ 50*
*k*-mer completeness	100.0%	*≥ 95%*
BUSCO **	C:95.3%[S:94.2%,D:1.1%], F:1.1%,M:3.6%,n:3,285	*C ≥ 95%*
Percentage of assembly mapped to chromosomes	97.26%	*≥ 95%*
Sex chromosomes	Not identified	*localised homologous pairs*
Organelles	Mitochondrial genome: 18.0 kb	*complete single alleles*
Genome annotation of assembly GCA_963082835.1 at Ensembl		
Number of protein-coding genes	10,816	
Number of non-coding genes	1,208	
Number of gene transcripts	17,910	

* Assembly metric benchmarks are adapted from column VGP-2020 of “Table 1: Proposed standards and metrics for defining genome assembly quality” from Rhie *et al.* (2021). ** BUSCO scores based on the diptera_odb10 BUSCO set using version 5.3.2. C = complete [S = single copy, D = duplicated], F = fragmented, M = missing, n = number of orthologues in comparison. A full set of BUSCO scores is available at https://blobtoolkit.genomehubs.org/view/Leptogaster%20cylindrica/dataset/CAUJAV01/busco.

**Table 3.** Chromosomal pseudomolecules in the genome assembly of *Leptogaster cylindrica*, idLepCyli1.

INSDC accession	Name	Length (Mb)	GC%
OY720105.1	1	51.73	36.0
OY720106.1	2	40.02	34.5
OY720107.1	3	39.82	35.0
OY720108.1	4	30.33	35.5
OY720109.1	5	29.32	35.5
OY720110.1	MT	0.02	22.5

The estimated Quality Value (QV) of the final assembly is 60.2 with *k*-mer completeness of 100.0%, and the assembly has a BUSCO v5.3.2 completeness of 95.3% (single = 94.2%, duplicated = 1.1%), using the diptera_odb10 reference set ( *n* = 3,285).

Metadata for specimens, BOLD barcode results, spectra estimates, sequencing runs, contaminants and pre-curation assembly statistics are given at https://links.tol.sanger.ac.uk/species/468752.

## Genome annotation report

The *Leptogaster cylindrica* genome assembly (GCA_963082835.1) was annotated at the European Bioinformatics Institute (EBI) on Ensembl Rapid Release. The resulting annotation includes 17,910 transcribed mRNAs from 10,816 protein-coding and 1,208 non-coding genes ( Table 2; https://rapid.ensembl.org/Leptogaster_cylindrica_GCA_963082835.1/Info/Index). The average transcript length is 6,777.07. There are 1.49 coding transcripts per gene and 5.88 exons per transcript.

## Methods

### Sample acquisition

An adult female *Leptogaster cylindrica* (specimen ID NHMUK014111064, ToLID idLepCyli1) was collected from Luton, England, UK (latitude 51.88, longitude –0.37) on 2020-06-17 by netting. The specimen used for RNA sequencing (specimen ID NHMUK014111078, ToLID idLepCyli3) was netted in the same location on 2020-06-23. The specimens were collected by Olga Sivell (Natural History Museum) and identified by Duncan Sivell (Natural History Museum) and preserved on dry ice.

The initial identification was verified by an additional DNA barcoding process according to the framework developed by Twyford *et al*. (2024). A small sample was dissected from the specimens and stored in ethanol, while the remaining parts of the specimen were shipped on dry ice to the Wellcome Sanger Institute (WSI). The tissue was lysed, the COI marker region was amplified by PCR, and amplicons were sequenced and compared to the BOLD database, confirming the species identification ( Crowley *et al.*, 2023). Following whole genome sequence generation, the relevant DNA barcode region is also used alongside the initial barcoding data for sample tracking at the WSI ( Twyford *et al.*, 2024). The standard operating procedures for Darwin Tree of Life barcoding have been deposited on protocols.io ( Beasley *et al.*, 2023).

### Nucleic acid extraction

The workflow for high molecular weight (HMW) DNA extraction at the Wellcome Sanger Institute (WSI) Tree of Life Core Laboratory includes a sequence of core procedures: sample preparation; sample homogenisation, DNA extraction, fragmentation, and clean-up. In sample preparation, the idLepCyli1 sample was weighed and dissected on dry ice ( Jay *et al.*, 2023). Tissue from the thorax was homogenised using a PowerMasher II tissue disruptor ( Denton *et al.*, 2023a). HMW DNA was extracted using the Manual MagAttract v1 protocol ( Strickland *et al.*, 2023b). DNA was sheared into an average fragment size of 12–20 kb in a Megaruptor 3 system with speed setting 30 ( Todorovic *et al.*, 2023). Sheared DNA was purified by solid-phase reversible immobilisation ( Strickland *et al.*, 2023a): in brief, the method employs AMPure PB beads to eliminate shorter fragments and concentrate the DNA. The concentration of the sheared and purified DNA was assessed using a Nanodrop spectrophotometer and Qubit Fluorometer using the Qubit dsDNA High Sensitivity Assay kit. Fragment size distribution was evaluated by running the sample on the FemtoPulse system.

RNA was extracted from thorax tissue of idLepCyli3 in the Tree of Life Laboratory at the WSI using the RNA Extraction: Automated MagMax™ *mir*Vana protocol ( do Amaral *et al.*, 2023). The RNA concentration was assessed using a Nanodrop spectrophotometer and a Qubit Fluorometer using the Qubit RNA Broad-Range Assay kit. Analysis of the integrity of the RNA was done using the Agilent RNA 6000 Pico Kit and Eukaryotic Total RNA assay.

Protocols developed by the WSI Tree of Life laboratory are publicly available on protocols.io ( Denton *et al.*, 2023b).

### Sequencing

Pacific Biosciences HiFi circular consensus DNA sequencing libraries were constructed according to the manufacturers’ instructions. DNA sequencing was performed by the Scientific Operations core at the WSI on a Pacific Biosciences Sequel IIe instrument. Hi-C data were also generated from head tissue of idLepCyli1 using the Arima-HiC v2 kit. The Hi-C sequencing was performed using paired-end sequencing with a read length of 150 bp on the Illumina NovaSeq 6000 instrument.

### Genome assembly, curation and evaluation

***Assembly***

The original assembly of HiFi reads was performed using Hifiasm ( Cheng *et al.*, 2021) with the --primary option. Haplotypic duplications were identified and removed with purge_dups ( Guan *et al.*, 2020). Hi-C reads are further mapped with bwa-mem2 ( Vasimuddin *et al.*, 2019) to the primary contigs, which are further scaffolded using the provided Hi-C data ( Rao *et al.*, 2014) in YaHS ( Zhou *et al.*, 2023) using the --break option. Scaffolded assemblies are evaluated using Gfastats ( Formenti *et al.*, 2022), BUSCO ( Manni *et al.*, 2021) and MERQURY.FK ( Rhie *et al.*, 2020).

The mitochondrial genome was assembled using MitoHiFi ( Uliano-Silva *et al.*, 2023), which runs MitoFinder ( Allio *et al.*, 2020) and uses these annotations to select the final mitochondrial contig and to ensure the general quality of the sequence.

***Assembly curation***

The assembly was decontaminated using the Assembly Screen for Cobionts and Contaminants (ASCC) pipeline (article in preparation). Manual curation was primarily conducted using PretextView ( Harry, 2022), with additional insights provided by JBrowse2 ( Diesh *et al.*, 2023) and HiGlass ( Kerpedjiev *et al.*, 2018). Scaffolds were visually inspected and corrected as described by Howe *et al*. (2021). Any identified contamination, missed joins, and mis-joins were corrected, and duplicate sequences were tagged and removed. The entire process is documented at https://gitlab.com/wtsi-grit/rapid-curation (article in preparation).

***Evaluation of the final assembly***

A Hi-C map for the final assembly was produced using bwa-mem2 ( Vasimuddin *et al.*, 2019) in the Cooler file format ( Abdennur & Mirny, 2020). To assess the assembly metrics, the *k*-mer completeness and QV consensus quality values were calculated in Merqury ( Rhie *et al.*, 2020). This work was done using the “sanger-tol/readmapping” ( Surana *et al.*, 2023a) and “sanger-tol/genomenote” ( Surana *et al.*, 2023b) pipelines. The genome readmapping pipelines were developed using the nf-core tooling ( Ewels *et al.*, 2020), use MultiQC ( Ewels *et al.*, 2016), and make extensive use of the Conda package manager, the Bioconda initiative ( Grüning *et al.*, 2018), the Biocontainers infrastructure ( da Veiga Leprevost *et al.*, 2017), and the Docker ( Merkel, 2014) and Singularity ( Kurtzer *et al.*, 2017) containerisation solutions. The genome was also analysed within the BlobToolKit environment ( Challis *et al.*, 2020) and BUSCO scores ( Manni *et al.*, 2021; Simão *et al.*, 2015) were calculated.

Table 4 contains a list of relevant software tool versions and sources.

**Table 4.** Software tools: versions and sources.

Software tool	Version	Source
BlobToolKit	4.2.1	https://github.com/blobtoolkit/blobtoolkit
BUSCO	5.3.2	https://gitlab.com/ezlab/busco
Hifiasm	0.16.1-r375	https://github.com/chhylp123/hifiasm
HiGlass	1.11.6	https://github.com/higlass/higlass
Merqury	MerquryFK	https://github.com/thegenemyers/MERQURY.FK
MitoHiFi	2	https://github.com/marcelauliano/MitoHiFi
PretextView	0.2	https://github.com/wtsi-hpag/PretextView
purge_dups	1.2.3	https://github.com/dfguan/purge_dups
sanger-tol/genomenote	v1.0	https://github.com/sanger-tol/genomenote
sanger-tol/readmapping	1.1.0	https://github.com/sanger-tol/readmapping/tree/1.1.0
YaHS	yahs-1.1.91eebc2	https://github.com/c-zhou/yahs

### Genome annotation

The Ensembl Genebuild annotation system ( Aken *et al.*, 2016) was used to generate annotation for the *Leptogaster cylindrica* assembly (GCA_963082835.1) in Ensembl Rapid Release at the EBI. Annotation was created primarily through alignment of transcriptomic data to the genome, with gap filling via protein-to-genome alignments of a select set of proteins from UniProt ( UniProt Consortium, 2019).

### Wellcome Sanger Institute – Legal and Governance

The materials that have contributed to this genome note have been supplied by a Darwin Tree of Life Partner. The submission of materials by a Darwin Tree of Life Partner is subject to the **‘Darwin Tree of Life Project Sampling Code of Practice’**, which can be found in full on the Darwin Tree of Life website here. By agreeing with and signing up to the Sampling Code of Practice, the Darwin Tree of Life Partner agrees they will meet the legal and ethical requirements and standards set out within this document in respect of all samples acquired for, and supplied to, the Darwin Tree of Life Project.

Further, the Wellcome Sanger Institute employs a process whereby due diligence is carried out proportionate to the nature of the materials themselves, and the circumstances under which they have been/are to be collected and provided for use. The purpose of this is to address and mitigate any potential legal and/or ethical implications of receipt and use of the materials as part of the research project, and to ensure that in doing so we align with best practice wherever possible. The overarching areas of consideration are:

•   Ethical review of provenance and sourcing of the material

•   Legality of collection, transfer and use (national and international)

Each transfer of samples is further undertaken according to a Research Collaboration Agreement or Material Transfer Agreement entered into by the Darwin Tree of Life Partner, Genome Research Limited (operating as the Wellcome Sanger Institute), and in some circumstances other Darwin Tree of Life collaborators.

## Data Availability

European Nucleotide Archive: *Leptogaster cylindrica*. Accession number PRJEB57890; https://identifiers.org/ena.embl/PRJEB57890 ( Wellcome Sanger Institute, 2023). The genome sequence is released openly for reuse. The *Leptogaster cylindrica* genome sequencing initiative is part of the Darwin Tree of Life (DToL) project. All raw sequence data and the assembly have been deposited in INSDC databases. Raw data and assembly accession identifiers are reported in Table 1 and Table 2.

## Author information

Members of the Natural History Museum Genome Acquisition Lab are listed here: https://doi.org/10.5281/zenodo.12159242.

Members of the Darwin Tree of Life Barcoding collective are listed here: https://doi.org/10.5281/zenodo.12158331

Members of the Wellcome Sanger Institute Tree of Life Management, Samples and Laboratory team are listed here: https://doi.org/10.5281/zenodo.12162482.

Members of Wellcome Sanger Institute Scientific Operations: Sequencing Operations are listed here: https://doi.org/10.5281/zenodo.12165051.

Members of the Wellcome Sanger Institute Tree of Life Core Informatics team are listed here: https://doi.org/10.5281/zenodo.12160324.

Members of the Tree of Life Core Informatics collective are listed here: https://doi.org/10.5281/zenodo.5013541.

Members of the Darwin Tree of Life Consortium are listed here: https://doi.org/10.5281/zenodo.4783558.
