# Supplementary material for: The genome sequence of a robberfly Leptogaster cylindrica (De Geer, 1776)
Source: Wellcome Open Res. 2024 Aug 12;9:443. [Version 1] doi: 10.12688/wellcomeopenres.22761.1 (PMC11480716; doi:10.12688/wellcomeopenres.22761.1)
Supplement: Peer review history

# Reviewer report: Reviewer response for version 1

**DOI:** 10.21956/wellcomeopenres.25065.r101039

**Published:** 2024-10-15

**Authors:** Maríndia Deprá

**Affiliations:** 1 Federal University of Rio Grande do Sul, Porto Alegre, State of Rio Grande do Sul, Brazil

The paper presents a high-quality genome assembly of *Leptogaster cylindrica*, derived from a female specimen. The assembly covers 196.60 MB, with most data scaffolded into five chromosomal pseudomolecules. Advanced sequencing techniques, such as PacBio HiFi long reads and Hi-C data, were employed, and a significant portion of the mitochondrial genome was sequenced. The study also identified 10,816 protein-coding genes, providing a valuable resource for future phylogenetic and evolutionary studies within the Asilidae family.

 The study is well-written and provides a comprehensive description of the genome assembly and sequencing. The methods used are appropriate, and the results are of high quality. The information presented will be valuable to researchers studying Asilidae phylogeny and biology.

 As a suggestion, maybe the researchers could explore alternative approaches to identifying sex chromosomes.

Are sufficient details of methods and materials provided to allow replication by others?

Yes

Is the rationale for creating the dataset(s) clearly described?

Yes

Are the datasets clearly presented in a useable and accessible format?

Yes

Are the protocols appropriate and is the work technically sound?

Yes

Reviewer Expertise:

Animal genetics with emphasis on insect evolutionary biology and transposable elements

I confirm that I have read this submission and believe that I have an appropriate level of expertise to confirm that it is of an acceptable scientific standard.

# Reviewer report: Reviewer response for version 1

**DOI:** 10.21956/wellcomeopenres.25065.r99022

**Published:** 2024-09-19

**Authors:** Guilherme B. Dias

**Affiliations:** 1 Department of Cell and Molecular Biology, Uppsala University, Uppsala, Uppsala Country, Sweden

This article describes the sequencing, assembly, and annotation of the genome of *Leptogaster cylindrica*, a robberfly from the Asilidae family, as part of the Tree of Life project.

 The authors used a single female individual for both HiFi and Hi-C sequencing, and the methods employed allowed separation of a principal chromosome-scale pseudohaplotype that can be used as a reference genome. A second female individual collected in the same location was used for obtaining RNA-seq data for gene annotation.

 The methods are well described and results seem to be of high quality according to EBP metrics. One small suggestion would be to include the SHA-hash prefix for the last commit in the MerquryFK version used in Table 4.

Are sufficient details of methods and materials provided to allow replication by others?

Yes

Is the rationale for creating the dataset(s) clearly described?

Yes

Are the datasets clearly presented in a useable and accessible format?

Yes

Are the protocols appropriate and is the work technically sound?

Yes

Reviewer Expertise:

Genome assembly; Genome annotation; Comparative Genomics

I confirm that I have read this submission and believe that I have an appropriate level of expertise to confirm that it is of an acceptable scientific standard.
